# Microenvironmental pH-Modulated Dissolution of Albendazole Layered on Tartaric Acid Starter Pellet Cores

**DOI:** 10.3390/pharmaceutics17091133

**Published:** 2025-08-29

**Authors:** Kristina Vlahovic, Miléna Lengyel, Christian Fleck, Nikolett Kállai-Szabó, Emese Balogh, András József Laki, István Antal

**Affiliations:** 1 Department of Pharmaceutics, Semmelweis University, Hőgyes E. Str. 7, 1092 Budapest, Hungary; vlahovic.kristina@phd.semmelweis.hu (K.V.); lengyel.milena@semmelweis.hu (M.L.); christian.petszulat@stud.semmelweis.hu (C.F.); kallai.nikolett@semmelweis.hu (N.K.-S.); balogh.emese@semmelweis.hu (E.B.); 2 Center for Pharmacology and Drug Research & Development, Semmelweis University, Üllői Str. 26, 1085 Budapest, Hungary; 3 Faculty of Information Technology and Bionics, Pázmány Péter Catholic University, Práter Str. 50/A, 1083 Budapest, Hungary; laki.andras.jozsef@itk.ppke.hu; 4 Department of Biophysics and Radiation Biology, Semmelweis University, Tűzoltó Str. 37–47, 1094 Budapest, Hungary

**Keywords:** multiparticulate, layered pellet, modified release, solubility enhancement, albendazole, inert core, functional core, tartaric acid, microenvironmental pH, drug repurposing

## Abstract

**Background/Objectives**: To improve the therapeutic efficacy of albendazole (ABZ) for ileocolonic diseases, its low solubility at higher pH levels should be enhanced. Organic acids have been widely used as pH modifiers to improve the solubility of weakly basic drugs. To achieve an adequate effect of the acidic microenvironmental pH during the drug release, pH modifiers should not leach out from the formulation. In the present work, we aimed to demonstrate that 100% tartaric acid pellets (TAP) can be used as pH-modifier cores, providing an acidic microenvironmental pH to enhance the solubility of albendazole at a higher pH. **Methods**: This study develops multilayer-coated pellets using TAP as starter cores in a bottom-spray-configured fluidized bed apparatus. The drug-layered TAP were coated with time-dependent and pH-dependent layers. **Results**: The release of ABZ from tartaric acid-based coated pellets was enhanced compared to that from pellets with the same layering structure but with an inert core of sugar or microcrystalline cellulose (MCC). In vitro experiments showed that tartaric acid remained in the pellet core during the dissolution test at a pH 6.8 medium, which resulted in an enhanced release of albendazole at a higher pH. The application of a combination of time-dependent and pH-dependent polymers aimed not only to prevent the release of albendazole at lower pH levels but also to protect TAP from premature release from the formulation. **Conclusions**: The application of 100% ready-to-use tartaric acid pellets (TAP) with the applied combination of coatings enhanced the solubility of the weakly basic drug albendazole at higher intestinal pH.

## 1. Introduction

Albendazole (ABZ) is a benzimidazole used as an anthelmintic drug in veterinary and human medicine [1]. Besides its antiparasitic use, the repurposing of ABZ has been investigated for cancer therapy [2]. It has potential as an anticancer agent against hepatocellular carcinoma [3] and ovarian cancer [4,5], and it exhibits an antiproliferative effect against acute lymphoblastic leukemia cell lines [6]. It was also investigated as a potential therapeutic strategy against skin cancer when combined with a T-LAK cell-originated protein kinase inhibitor [7]. Furthermore, it carries a strong potential to act against colon cancer [8]. Moreover, it has been demonstrated that ABZ enhances the clinical efficacy of anti-TNF (tumor necrosis factor) therapy in inflammatory bowel diseases [9]. However, the low solubility of ABZ should be increased to achieve higher therapeutic efficacy for colon diseases [10].

A significant challenge in formulating this active pharmaceutical ingredient (API) lies in its low solubility and bioavailability. In the BCS (Biopharmaceutical Classification System), ABZ is classified as a class II/IV substance characterized by low solubility and high/low permeability [11]. It is a weak basic compound with pH-dependent solubility of 0.016 mg/mL in a pH = 6.0 buffer solution [12]. The solubility in the acidic medium (pH = 1.2) was found to be 1.520 mg/mL by Fülöp et al. [13], and 0.376 mg/mL by Torrado et al. in a previous study [12]. Furthermore, the absorption of ABZ was expected to be higher in the gastric region than in the intestine [14,15], which would reduce the amount of available unabsorbed drug concentration in the lower parts of the gastrointestinal tract. As ABZ is directly active against intestinal helminths [16], and due to its repurposing for other colon diseases, a higher concentration of ABZ in the lower parts of the gastrointestinal tract is preferable for a better therapeutic effect. However, a study suggested that certain doses of ABZ sulfoxide may be responsible for fetal toxicity in animals [17]. Other studies show that the doses of 10 mg per kg per day used in rabbits and doses of 6.6 mg and 8.8 mg/kg/day and above for rats produced developmental toxicity in these animals [18].

In contrast, no developmental toxicity was observed in rabbits, rats, and dogs at a dose of 5 mg/kg/day [18]. To mitigate the toxic effects of higher doses, the local targeting of ABZ may provide an opportunity to decrease the dose required to achieve anthelminthic activity [19]. In the case of colon cancer, local delivery and improved solubility of albendazole reduced the half-maximal inhibitory concentration (IC50) in colorectal cancer cell lines [10].

Various approaches have been employed to enhance the solubility of albendazole by forming salts with different acids [20], formulation of solid dispersions [21], pH-sensitive solid dispersions [22], nanosuspensions [10,13], and self-microemulsifying drug delivery systems in chewable tablets [23]. Multiparticulate dosage forms, ABZ-loaded Avicel^®^ pellets, were formulated with different hydrophilic agents to enhance ABZ solubility [24]. Another approach to improve the solubility of weakly basic drugs is the use of pH modifiers in extended-release dosage forms. Pareek et al. showed that InstaSpheres TA (tartaric acid spheres seal-coated with hydrophilic polymer—Ideal Cures, Mumbai, India) provide pH modulation for the controlled release of the weakly basic drug dipyridamole at a higher pH, despite high solubility and the low acidic strength of tartaric acid [25].

In cases of diseases that affect the colon, targeted delivery of the API to the colon is rational, as it aims to target the release of the drug and thereby reduce adverse systemic effects while increasing the bioavailability of the drug [26]. Su et al. combined the enhancement of the solubility with the targeted delivery to maximize the release of ABZ in the enteric region, where the drug solubility was increased by the formulation of pH-sensitive solid dispersions with the pH-sensitive solubilizing agent hypromellose acetate succinate [22]. Guo et al. also combined the improvement of albendazole solubility by nanosuspension with the colon-delivery EUDRACAP^®^-based formulation [10]. The increase in the low solubility of albendazole at the targeted site with a higher pH can potentially lead to a higher bioavailability.

Among various techniques, Eudragit^®^ FS (EuFS) polymer has been used for the targeted delivery of drugs due to its pH-dependent solubility (soluble above pH 7) [27]. Additionally, it has already been used to control the release of a low-soluble API [28].

We have formulated a modified-release multiparticulate dosage form using functional pellet cores of solely 100% tartaric acid–tartaric acid pellets (TAP). Eudragit^®^ FS was used to provide targeted delivery of ABZ to higher pH levels, thereby preventing its release in the gastric region. For a more reliable release, Eudragit^®^ RS (EuRS) or Eudragit^®^ RL (EuRL) type was used as a time-dependent polymer. The hypothesis was that at the target site of higher pH, solubilization enhancement by functional pH-modifier cores would increase the local solubility of ABZ. As hydrophobic polymers are generally not suitable for low-soluble substances [29,30], the aim was to use Eudragit^®^ RS in combination with TAP, which was supposed to improve ABZ solubility. The release of ABZ from tartaric acid-based drug-loaded polymer-coated pellets was compared to that from pellets with the same layering structure but using an inert core of sugar or microcrystalline cellulose (MCC). To follow the pH changes inside the pellet core, the microenvironmental pH was studied using the slurry method, where the pH of the supernatant from a slurry of TAP with different coating layers was compared. The color change induced by the dissolution of tartaric acid from coated pellets was also observed in the microfluidic cell, further confirming the lowering effect of the microenvironmental pH by tartaric acid and demonstrating the different dissolution behaviors of tartaric acid from time-dependent and pH-dependent coating layers. The final formulation was designed to prevent the release of ABZ at lower pH levels, thereby maximizing drug release in the alkaline intestinal region and enhancing solubility through functional pellet cores.

## 2. Materials and Methods

### 2.1. Materials

Albendazole EP (micronized) (SeQuent Scientific Ltd., Bilekahalli, India) was used as a model drug. TAP 700 (600–800 µm; IPC Process-Center GmbH, Dresden, Germany, Harke Pharma GmbH, Mülheim an der Ruhr, Germany) was used as a pH-modifier core. Sugar spheres (710–850 µm, pharm-a-spheres^®^, H.G.Werner GmbH, Tornesch, Germany; and 500–710 µm, Ethispheres^®^ 600, NPPharm Ltd., Bazainville, France) were chosen as cores for comparison with TAP. Hydroxypropyl methylcellulose (HPMC; Pharmacoat 606, Shin-Etsu Chemical Ltd., Tokyo, Japan) was used as a binder for the ABZ-layering process. Polymethacrylate copolymers (Eudragit^®^ RL30D, Eudragit^®^ RS30D, and Eudragit^®^ FS30D, Evonik, Essen, Germany)—abbreviated as EuRL, EuRS, and EuFS, respectively—were used as film-forming polymers. Triethyl citrate (TEC; Fluka Chemie AG, Buchs, Switzerland) served as a plasticizer, and micronized talc (Sigma-Aldrich Chemie GmbH, Taufkirchen, Germany) was used as an anti-adhesion agent.

### 2.2. Coating Process

#### 2.2.1. Drug Layering of Inert Cores

ABZ (10.0% *w*/*w*) was dispersed in HPMC (Pharmacoat 606; 2.0% *w*/*w*) liquid with the overhead stirrer (Hei-TORQUE, Heidolph Instruments, Schwabach, Germany) at rpm 840. The suspension was then layered onto the different types of cores in a bottom spray-configured fluidized bed apparatus with nozzles possessing diameters of 0.8 mm and 1.2 mm (Aeromatic Strea I., Aeromatic-Fielder AG, Bubendorf, Switzerland); 10.0% *w*/*w* drug concentration was achieved. The dispersion was stirred continuously during layering to maintain a homogeneous dispersion of the API. The process parameters are summarized in Table 1.

#### 2.2.2. Coating of Drug-Layered Pellets

**Figure 1:**
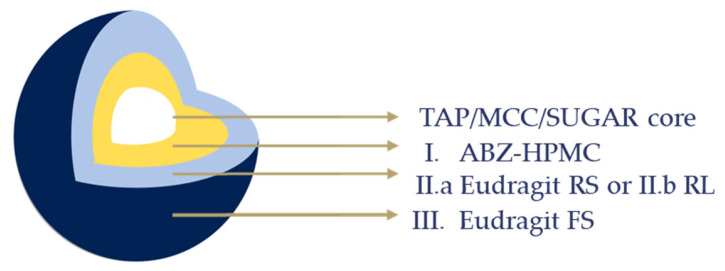
Schematic representation of the multilayer enteric-coated pellet for modified-release.

The ABZ-layered TAP, sugar, and MCC cores were coated with different time-dependent polymers, EuRS or EuRL (second layer), and a pH-dependent polymer, EuFS (third layer; Figure 1). TEC was used at a concentration of 20% *w*/*w* on dry polymer for EuRL and EuRS, and 10% *w*/*w* on dry polymer for EuFS. Talc was included in the coating composition at 75% and 55% *w*/*w* concentrations of the dry polymer in the case of EuRS or EuRL and EuFS, respectively. The concentration of EuRS and EuRL polymers was 10% *w*/*w*, and that of EuFS was 13.65% *w*/*w* in the coating suspension. The dispersions were gently and continuously stirred during the coating processes to prevent sedimentation of the talc. The process parameters are listed in Table 1, which were selected according to the previous studies [31,32,33]. The TAP–ABZ ratio in the final product is 30:1, the drug loading is 2.1% *w*/*w*.

The coated beads were stored in tightly closed containers after the coating process. The dissolution studies were performed three days after the coating process (within 2 weeks). All results are the mean of three parallels.

### 2.3. Shape and Size of the Pellets

One hundred eighty pellets were randomly chosen from each batch to be analyzed. The pellets were placed on the black background plate of the digital microscope (Keyence VHX-970F; lens, Z20:X20; Keyence Corp., Osaka, Japan). A maximum of 15–30 pellets could be photographed at a time. Each batch was photographed using 6–12 photos, and the images produced were analyzed using the computer program ImageJ (v.1.54g), a freely available software for image analysis (National Institutes of Health, Bethesda, MD, USA). In this study, the pellet size and shape were characterized by aspect ratio (AR), Feret diameter (ImageJ), and equivalent spherical diameter (d_eq_) calculated from the formula of the projected area of a sphere [34]:

(1) $$d_{\mathrm{eq}}\;=\;\sqrt{\frac{4A\;}{\pi }\;}$$

Roundness (R) was calculated using the following formula [34]:

(2) $$R=\frac{p^{2}}{4\pi A},$$

where p is the perimeter, and A is the area of the pellet. Aspect ratio (AR) is the ratio of the maximum Feret diameter (Feret_max_) to the minimum Feret diameter (Feret_min_), perpendicular to the maximum Feret diameter. The values presented for each type of pellet are the average and standard deviation (SD), which were calculated from measurements of 180 individual pellets.

### 2.4. Scanning Electron Microscope (SEM) Imaging

The Scanning Electron Microscope images of the cross-section of coated pellets were taken with a Jeol JSM-5200 Scanning Probe Microscope (Jeol Ltd., Tokyo, Japan) at 15 kV. Pellet samples were split in half by a razor blade while held with tweezers to enable cross-sectional visualization. Samples were fixed on a graphene tape on a Cu/Zn sample holder. Graphene and gold coating were used in the sample-preparation procedure.

### 2.5. Thermodynamic Solubility Determination

An excess amount of ABZ powder (15 mg) was mixed with different buffers (0.1 N hydrochloric acid-pH 1.2, 0.05 M phosphate buffer solution pH 4.5, phosphate buffer-pH 6.8, and phosphate buffer solution pH 7.2; Ph. Eur. 10). To investigate the increase in solubility in phosphate buffer pH 6.8 in the presence of TAP, the ratio in the TAP–ABZ mixture varied: 3.3:1.0, 5.0:1.0, 6.7:1.0, 12.5:1.0, 25:1.0, 50:1.0, 75:1.0, and 100:1.0. ABZ and TAP were poured into volumetric flasks of 20 mL capacity. The volumetric flasks were mixed using a heatable magnetic stirrer MS-H-S10 (DLAB Instruments Ltd., Beijing, China) and magnetic stirrer bars at 25 ± 0.5 °C for 24 h. The samples containing ABZ suspensions were filtered with a 0.22 µm pore-sized hydrophilic PTFE syringe membrane filter (Labex FilterBio Membrane Co. Ltd., Nantong, China). The amount of ABZ was calculated from the linear calibrations in each dissolution medium, based on three parallel measurements, and was determined by spectrophotometry at the absorption maximum of albendazole at a wavelength of λ_max_ = 295 nm using an Agilent 8453 spectrophotometer (Agilent Technologies Inc., Santa Monica, CA, USA). The pH was measured by a Mettler-Toledo Seven Compact micro pH/Ion meter (Mettler-Toledo International Inc., Columbus, OH, USA). The sample size was 500 µL. The pharmacopeial buffers had a nominal pH of ±0.05.

### 2.6. In Vitro Drug Release Test

The dissolution test for each batch of coated pellets was carried out in a 900 mL dissolution medium of pharmacopeial compositions (Ph. Eur. 10) pH 1.2 (0.1 N hydrochloric acid solution) for 2 h, in phosphate buffer pH 6.8 for the next 4 h, and finally in buffer solution pH 7.2 (phosphate buffer) further up to 24 h, according to pharmacopeial instructions [35] and the method developed by Zhang et al. for colon delivery Eudragit^®^ FS-coated granules [27]. The USP basket method with 100 rpm at 37 ± 0.5 °C was used (Hanson SR8-Plus™ Dissolution Test Station, Teledyne Hanson Research, Chatsworth, CA, USA). At predetermined time points, 5 mL samples were withdrawn. The concentration of released albendazole was measured (λ_max_ = 295 nm; Agilent 8453 spectrophotometer (Agilent Technologies Inc., Santa Monica, CA, USA) spectrophotometrically.

### 2.7. Microenvironmental pH Studies (Slurry Method)

Microenvironmental pH was studied using the slurry method [36]. The pH of the slurry is intended to represent the microenvironmental pH [36,37]. The dissolution test was conducted in a 900 mL phosphate buffer, pH 6.8, to compare the pH-modifying effect of TAP in the presence of different coatings. The samples were taken at 30 min, 1 h, 2 h, and 4 h. The batches used for microenvironmental pH studies were tartaric acid-based drug-layered pellets coated with different types of polymers (EuRS only and the combination of EuFS applied on EuRS). The internal content remaining in the pellet was obtained by crushing it in a mortar with a pestle. The slurries of crushed pellets were obtained by adding 1.35 mL of dissolution medium, pH 6.8. The slurries were then allowed to settle for 24 h, after which the pH of the supernatant was measured using a Mettler-Toledo Seven Compact micro pH/Ion meter (Mettler-Toledo International Inc., Columbus, OH, USA). The sample size was 200 µL. All results were the mean of three parallel samples (SD < 5%).

### 2.8. The Investigation of Release of Tartaric Acid in the Microfluidic Setup Based on Image Analysis and CIELab Measurement

The experiments were conducted using a microfluidic device tailor-made via soft lithography (Microfluidic dissolution tester by Laki Technology (BioMicrofluidics Lab PPKE ITK, Budapest, Hungary). The medium flew with a low flow rate (4000 µL/h) through the micro-scale channels, and the pellets were observed under the microscope (Nikon SMZ, Nikon, Tokyo, Japan); 1000 Optics 1×, Magn: 3×), with photographs taken at pre-set times. A similar microfluidic testing device was used for release tests by Amoyav et al. and Ren et al. [38,39]. This study aimed to observe and evaluate the release of tartaric acid from different coating layers of TAP-based cores at a pH of 6.8, thereby explaining the in vitro drug release and microenvironmental pH alterations within the pellet. The indicator methyl red (Merck, Sigma-Aldrich Chemie GmbH, Taufkirchen, Germany) was used to show the dissolution of tartaric acid. The color change seen in the indicator in both the pellet core and the surrounding dissolution medium was studied in a microfluidic setup. The parameters of the CIELab color space (L*, a*, and b*) were determined at multiple points of each pellet and the dissolution medium at pre-set times. The difference in each color parameter is calculated from the actual minus the initial value according to the following equation (where X represents L*, a*, or b*):

(3) $$\Delta X\;=\;X_{\mathrm{actual}}-X_{\mathrm{initial}}$$

Meanwhile, the difference in the color change (${\Delta E}^{*}$) is calculated based on the following equation:

(4) $${\;\Delta E}^{*}=\sqrt[{}]{{{\Delta L}^{*}}^{2}+{{\Delta a}^{*}}^{2}+{{\Delta b}^{*}}^{2}}$$

To show the relationship between the color change (${\Delta E}^{*}$) and the ABZ release, parallel drug-release studies were performed at pH 6.8. To examine the relationship between the two datasets (cumulative drug release (%) and ${\Delta E}^{*}),$ a scatterplot was generated to assess the pattern and direction of the relationship visually. Additionally, Spearman’s correlation coefficient was calculated to quantify the strength of the monotonic correlation according to the following formula:

(5) $$r\;=\;1-\frac{6\Sigma d^{2}}{n\left(n^{2}-1\right)},$$

where d is the difference between the two ranks of each observation, and n is the number of observations.

The cumulative drug release (%) was attributed to x values, while the ΔE* was attributed to y values. Spearman’s correlation coefficient, ranging from 0.8 to 1.0, indicates a very strong correlation [40].

### 2.9. Osmolality Studies

We determined the osmolality of the three different pellet cores, TAP, MCC, and sugar spheres to compare the osmotic effect they might exert during the release process. Each pellet core was weighed accurately (7 mg ± 0.1 mg) and then soaked in 100 µL of freshly prepared distilled water. After the cores of TAP and sucrose from sugar spheres were dissolved entirely, a 60 µL sample was analyzed using a Gonotec Osmomat 030 (Gonotec GmbH, Berlin, Germany). As MCC cores generally do not disintegrate, after soaking in water for a specific time, they were centrifuged in a Sorvall TC6 laboratory centrifuge (DuPont, Fort Wayne, IN, USA) at 3000 rpm for 10 min. A 60 µL centrifuged sample was analyzed with Gonotech Osmomat 030. The results are the mean of six parallels.

## 3. Results

### 3.1. Morphological Properties of Pellets

**Figure 2:**
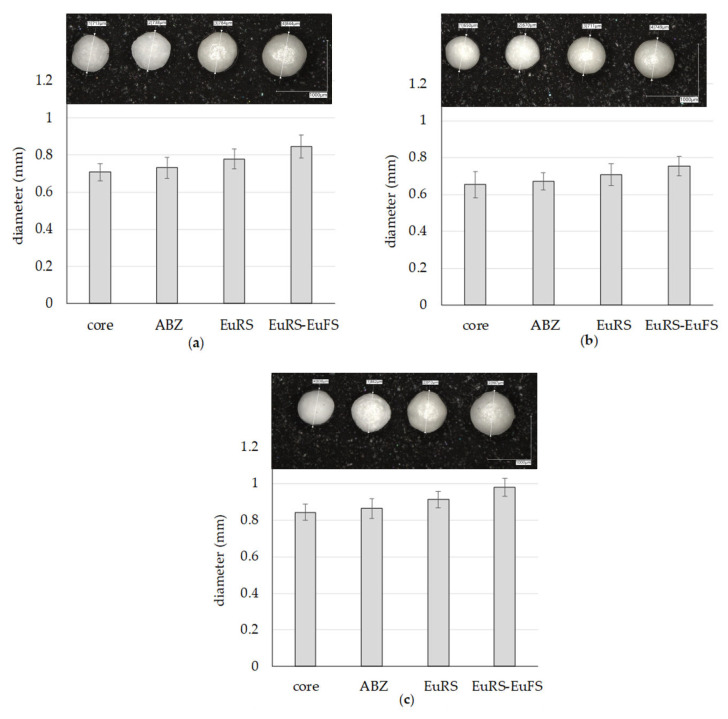
Increase in the pellet diameter, with the addition of the three layers: ABZ (HPMC) layer, Eudragit RS10% (EuRS) and Eudragit^®^ FS25% (EuFS) on (**a**) TAP, (**b**) MCC, and (**c**) sugar cores.

The Feret_max_, Feret_min_, and diameter values increase with the addition of the drug layer and coating layers to approximately the same extent for TAP and sugar pellets (Figure 2a,c, Supplementary Materials Table S1). In the case of MCC pellets with a smaller particle size, the coating layers were thinner than for TAP and sugar pellets (Figure 2b). The average roundness value for TAP is around 0.87 ± 0.06, which indicates a less spherical shape of tartaric acid pellets than sugar or MCC pellets (for those cores, roundness is 0.91 ± 0.06 and 0.92 ± 0.05, respectively; Supplementary Materials Table S1) and is further confirmed by microscopic pictures (Figure 2a–c). The roundness of the initial TAP cores was improved during the coating process, as confirmed by microscopic images.

**Figure 3:**
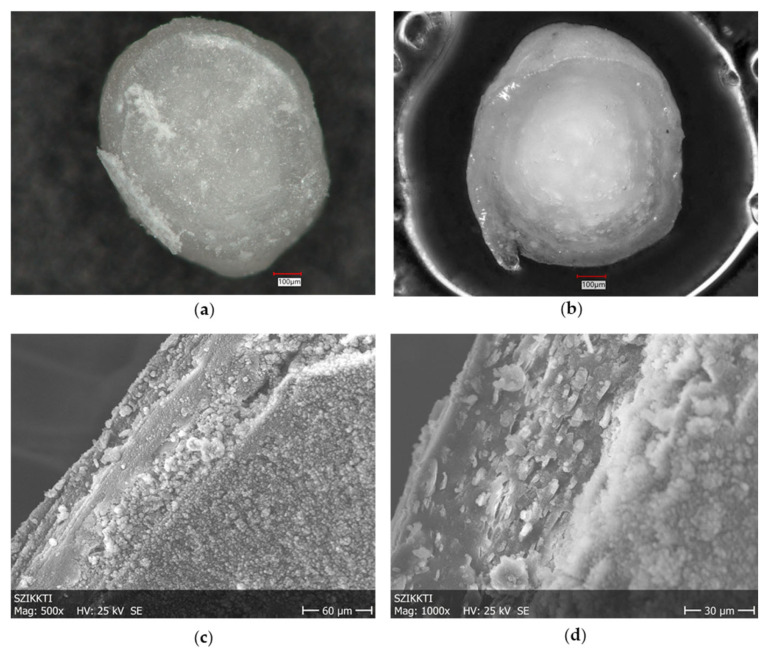
Cross-sections of TAP-EuRS10%-EuFS25% pellets: (**a**) dry structure, (**b**) studied in buffer solution pH 7.2 (t = 5 min) (Keyence VHX 970F digital microscope, Keyence Corp., Osaka, Japan), and (**c**) SEM (Jeol JSPM-5200 Scanning Probe Microscope (Jeol Ltd., Tokyo, Japan). (**d**) Cross-section of Sugar-EuRS10%-EuFS25% pellet by SEM.

Microscopic and SEM images of the cross-sections of the pellets in the dry state show the morphological characteristics of the pellets, which are even more expressed during the release process of the coated pellets (Figure 3).

### 3.2. Thermodynamic Solubility

**Figure 4:**
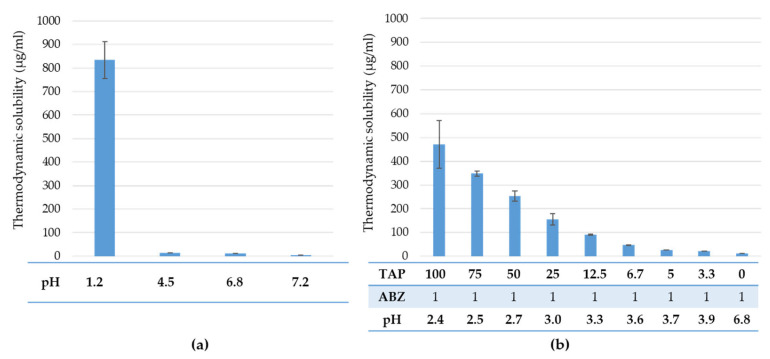
Thermodynamic solubility of ABZ powder in (**a**) different standardized dissolution media (pH 1.2, 6.8, 4.5 and 7.2) (Ph. Eur. 10) and (**b**) phosphate buffer at pH 6.8 with varying mass ratios of TAP-ABZ (100:1.0, 75:1.0, 50:1.0, 25:1.0, 12.5:1.0, 6.7:1.0, 5.0:1.0, 3.3:1.0, 0:1.0), at 25 ± 0.5 °C.

As previously described in studies, ABZ exhibits pH-dependent poor water solubility [12,13]. The lowest value of ABZ solubility was registered at pH = 6.8 (12.21 ± 0.24 µg/mL) and pH = 7.2 (4.61 ± 0.19 µg/mL) due to the dominance of ABZ unionized form [14], while the maximal solubility of ABZ powder was at pH 1.2 (834.47 ± 79 µg/mL). To maintain a consistent buffer strength, the thermodynamic solubility was measured in a pH 6.8 buffer solution (Ph.Eur.) at 25 ± 0.5 °C, using various mass ratios of TAP to ABZ (100:1.0, 75:1.0, 50:1.0, 25:1.0, 12.5:1.0, 6.7:1.0, 5.0:1.0, 3.3:1.0, and 0:1.0). During the process, the pH of the dissolution medium was monitored, as it varied according to the different TAP–ABZ ratios. The presence of TAP in pH 6.8 buffer solution decreased the pH and increased the solubility of ABZ due to the cationic form of ABZ [14]. Our measurement confirmed that, depending on the TAP–ABZ ratio, the ABZ solubility can be influenced, with a higher value observed at higher TAP concentrations (Figure 4b). The solubility of ABZ in pH 6.8 in the presence of TAP with different ratios, namely TAP–ABZ 12.5:1.0, TAP–ABZ 25:1.0, TAP–ABZ 50:1.0, TAP–ABZ 75:1.0, and TAP–ABZ 100:1.0 weight, was increased to 90.66 ± 1.99 µg/mL, 155.65 ± 4.09 µg/mL, 252.80 ± 21.09 µg/mL, 348.00 ± 11.33 µg/mL, and 470.70 ± 8.20 µg/mL respectively, suggesting that the presence of tartaric acid successfully improved the ABZ solubility.

### 3.3. In Vitro Drug Release

**Figure 5:**
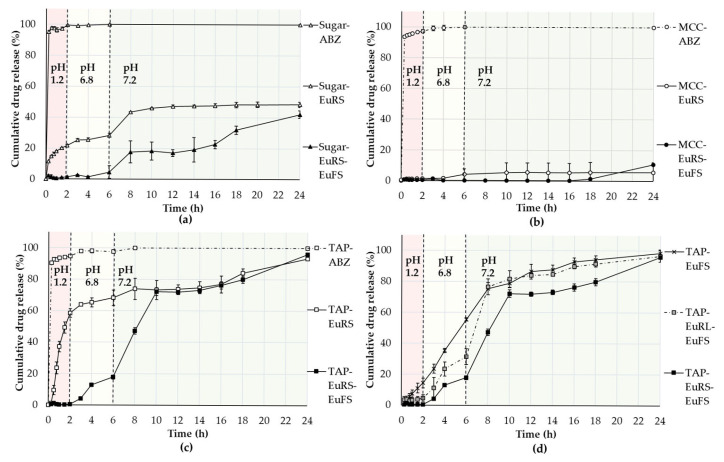
In vitro drug release of drug layered pellets (ABZ), EuRS 10% layer (EuRS), and EuRS10%-EuFS25% (EuRS-EuFS) on (**a**) sugar-based pellets, (**b**) MCC-based pellets, and (**c**) TAP. (**d**) Comparison of the release from TAP coated with EuRL10%FS25% (EuRL-EuFS), EuRS10%FS25% (EuRS-EuFS), and EuFS25% (EuFS) (USP basket method, 100 rpm; 900 mL medium at 37 °C; 0–2 h in pH 1.2; 2–6 h at pH 6.8; 6–24 h at pH 7.2).

The ABZ release was considered complete from the ABZ-layered pellets within the first 15 min, regardless of the core material (Figure 5a–c), which is attributed to the pH-dependent solubility of ABZ (Figure 4). However, once the EuRS 10% was layered on the surface, TAP pellets enhanced the release compared to other non-pH-modifier cores (Figure 5c).

In comparison with the time-dependent layer EuRS, the application of a pH-dependent polymer coating layer on top of the time-dependent one prevented the early release of ABZ at a lower pH (Figure 5a–c). The cumulative drug release (%) from enteric-coated pellets in the first 2 h at pH 1.2 was less than 1%. By TAP, after the pH change from 1.2 to 6.8 at 2 h, the ABZ release began to increase slightly over the next 4 h, despite EuFS generally being insoluble at pH 6.8 (Figure 5c). The same dissolution release performance with EuFS was previously observed by Zhang et al. [27].

After the pH changed to 7.2 at 6 h, the release rate increased rapidly, and nearly 100% of the drug was released from TAP-layered cores within 24 h (Figure 5c). In the case of both the time-dependent layer and the combination of time- and pH-dependent layers, the release resulted in being higher from TAP-coated pellets, compared to MCC cores and sugar cores (Figure 5a–c). However, the pH-modifying effect of TAP appears to be more efficient when the enteric coating EuFS is applied, suggesting that the presence of EuFS not only decreases the release of the drug at pH levels lower than 7 but also prevents tartaric acid from leaching out of the formulation at these pH levels. Therefore, the acidic microenvironmental pH is expected to be maintained, which is necessary for enhancing ABZ solubility and release at a higher pH.

The release from MCC-coated pellets, both with EuRS and EuFS, remained very low during the 24 h (Figure 5b) due to the water insolubility of the cores [31].

Furthermore, we aimed to compare the effect of time-dependent polymers of varying permeability on the drug release from enteric-coated TAP and to compare it with the pH-dependent layer applied alone. Within the first 8 h, the use of EuRS resulted in a more pronounced delayed release compared to EuRL in the enteric-coated formulations, preventing the release of albendazole more efficiently at pH levels lower than 7. After 6 h, the cumulative release was around 55%, 34%, and 18%, using EuFS, EuRL-EuFS, and EuRS-EuFS, respectively (Figure 5d).

### 3.4. Microenvironmental pH Study

**Figure 6:**
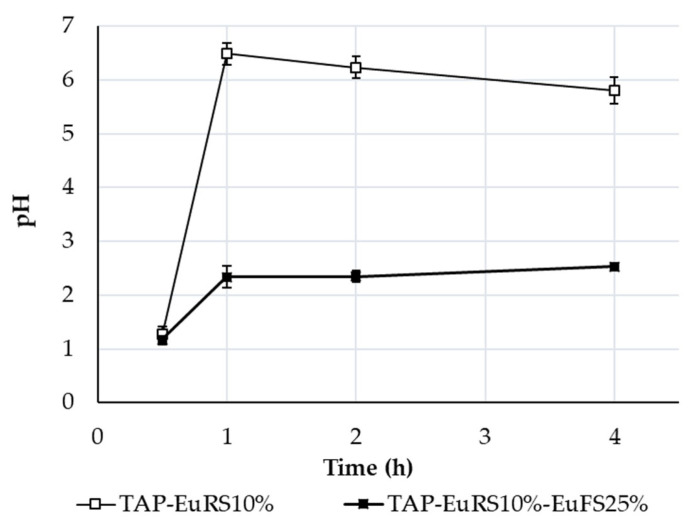
Microenvironmental pH studies conducted over 4 h at pH 6.8.

Microenvironmental pH studies were conducted to investigate the potential of the pH-modifying effect of TAP at pH 6.8 in enteric-coated pellets. A further objective was to confirm the hypothesis that the pH-modifying effect of tartaric acid in TAP-EuRS10%-EuFS25% is more efficient than in TAP-EuRS10%, due to its longer presence in the coated pellet, which may explain the release summarized in Figure 5. The results show that the pH of the supernatant of the slurry of TAP-EuRS10%-EuFS25% is more acidic than for TAP-EuRS10% (Figure 6). When the EuFS coating layer is applied in combination with a time-dependent layer, tartaric acid is preserved and does not leach out of the formulation at a pH of 6.8.

The pH of the slurry remains acidic throughout the 4 h in the case of EuRS-EuFS pellets, indicating the presence of TAP as a pH modifier, which increases solubility and leads to a higher release of ABZ compared to sugar and MCC cores (Figure 5a–c). Tartaric acid has two pKa values: 2.93 and 4.23 (at 25 °C) for the first and second carboxylic groups [41]. The pH of the slurry of TAP-EuRS10%-EuFS25% rose to 2.53 after 4 h of the study, approaching the first pKa value of the tartaric acid (2.93), because as time passes, the dissolution medium dissolves a part of the tartaric acid inside the pellet, making the pH of the slurry less acidic and closer to the pKa value.

### 3.5. The Release of Tartaric Acid from Enteric-Coated Pellets

**Figure 7:**
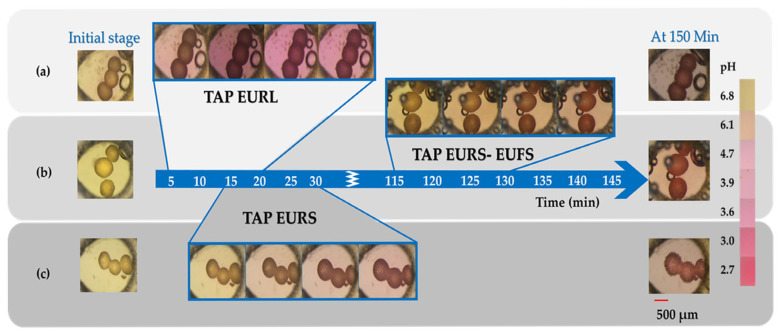
Observation of tartaric acid dissolution and release in the microfluidic setup at pH 6.8, followed by the color change seen in the methyl red indicator. The color turns red due to an acidic microenvironmental pH in the pellet cores and, as a result of dissolution, around the core in the external dissolution medium. The figure demonstrates the 15 min intervals where a remarkable color change (ΔE* > 5) was detected for TAP-EuRL10%, TAP-EuRS10%, and TAP-EuRS10%-EuFS25% pellets (medium flow: 4000 µL/h), under a microscope (Nikon SMZ 1000 Optics 1×, Magn: 3×). On the left- and right-hand sides, the demonstrative images taken at the start of the examination and after 150 min are shown from the top to the bottom: (**a**) TAP-EuRL10%, (**b**) TAP-EuRS10% FS25%, and (**c**) TAP-EuRS10%, respectively. The color–pH range scale is for an approximation of the conditions.

At an initial pH of 6.8, methyl red had a yellow color. As the release medium penetrated the coating layers and the tartaric acid in the cores began to dissolve, the lower pH in the center of the coated pellet was visualized by the indicator turning red. This was a quicker process by EuRS, starting within the first 30 min (Figure 7).

The observed color change in the indicator demonstrates its diffusion through the coating layer and subsequent entry into the core, where the indicator exhibits a red coloration in response to the acidic pH generated by tartaric acid dissolution. This transformation is initiated more rapidly in EuRL cores, attributable to their higher coating permeability compared to EuRS-coated pellets, which possess a less permeable functional layer. In the case where the EuFS layer was applied on top of EuRS, the color change was delayed, reaching a visually observable shift in color (ΔE* > 5) in the pellets after approximately 2–3 h (Figure 7). The color change is observed both in the pellet core and the surrounding medium as it comes to the time-dependent polymer, EuRS. In contrast, in the case of TAP-EuRS10%-EuFS25% layered pellets, the color change was more pronounced in the center of the pellet core than in the medium (Figure 7). This further confirms the previously stated hypothesis that tartaric acid is not easily released from the EuFS coating at pH levels lower than 7, thereby providing an acidic microenvironmental pH inside the pellet and, consequently, facilitating higher drug release.

**Figure 8:**
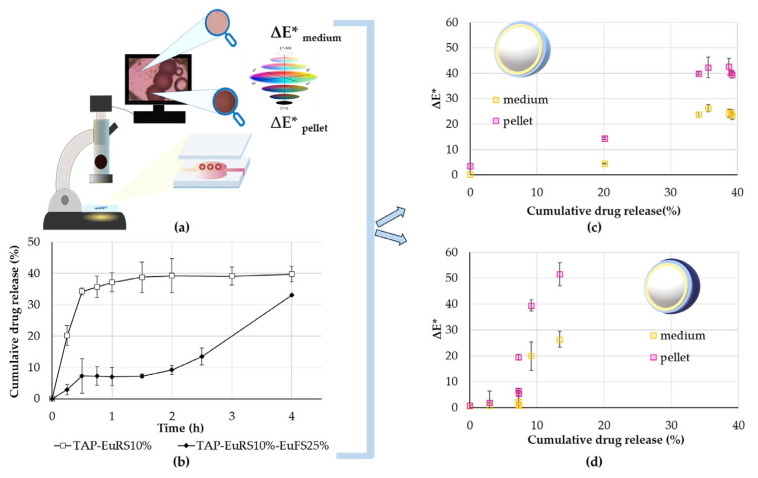
(**a**) Schematic image of the calculation of color change (ΔE*) by CIELab color space of the core and the dissolution media due to the microenvironmental pH change caused by tartaric acid dissolution and release indicated by methyl red. The relative change was always compared to the initial CIELab values of the medium measured at time point zero and of the pellets measured at time point zero and was the result of parallel measurements. (**b**) The cumulative release values of ABZ in the case of TAP pellets (TAP-EuRS10% and TAP-EuRS10%-EuFS25%). The release was tested in a pH 6.8 phosphate medium. (**c**,**d**) Relationship between ΔE* (medium and pellet) and the respective % drug release at specific time points by (**c**) TAP-EuRS10% and (**d**) TAP-EuRS10%-EuFS25%.

The color change and release values of the TAP EuRS10%-EuFS25% and TAP-EuRS10% pellets were compared to find a correlation between the microenvironmental pH changes and the release of ABZ from coated TAP cores (Figure 8a,b). For both measurements, a pH 6.8 phosphate buffer medium was used.

The scatterplots in Figure 8 illustrate how ΔE* (pellet) and ΔE* (medium) vary with the cumulative drug release (%) at pH 6.8. Both scatterplots (Figure 8c,d) reveal a trend where ΔE* values in the pellet core generally increase alongside the cumulative drug release (%), indicating a positive correlation between these variables at corresponding time points. To quantify the strength of the correlation between these two variables, the Spearman correlation coefficient is calculated and shown in Table 2.

The Spearman coefficient above 0.8 indicates a strong positive monotonic correlation [40]. However, the increase in the ΔE* values in the medium by TAP-EuRS10%-EuFS25% is not as pronounced as it is in the core (Figure 8d), which is in accordance with the microfluidic study (Figure 7). The scatterplot of the EuRS coating layer applied alone (Figure 8c) exhibits a distinct pattern compared to the EuRS-EuFS scatterplot (Figure 8d) and is characterized by a tight aggregation of data points within a narrow range after the first 30 min. In this case (Figure 8c), a significant color change was observed within the first 30 min, with minimal variability afterwards, both in the medium and the pellet core, which suggests the rapid process of TAP being dissolved and getting released from the EuRS-coated pellet (Supplementary Materials Table S2).

### 3.6. Osmolality Study

**Figure 9:**
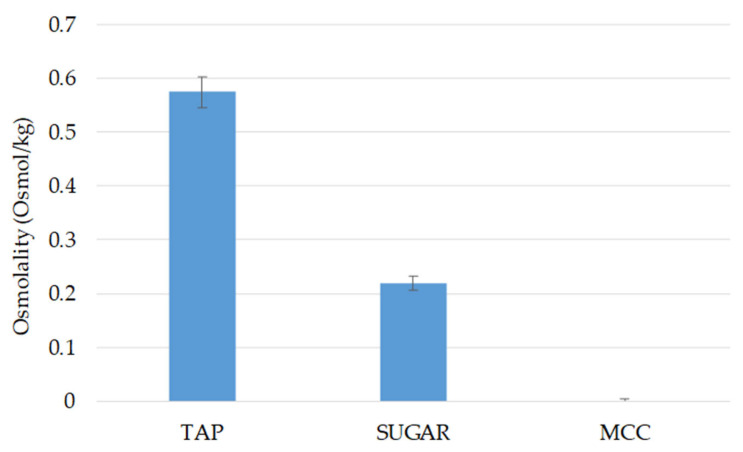
Osmolality (Osmol/kg) of TAP, sugar, and MCC pellet core-type solutions.

The osmolality (Osmol/kg) of the different pellet core solutions was determined as described previously. Among the pellet cores tested, the TAP solution exhibited the highest osmolality, measured at approximately 0.570 Osmol/kg (Figure 9). In comparison, the solution of sugar spheres exhibited lower osmolality, approximately one-third of the TAP osmolality, while the water-insoluble MCC led to an osmolality of 0, as expected.

The higher osmolality of TAP suggests that it may influence in vitro release studies, in addition to the effect of microenvironmental pH [42].

## 4. Discussion

As ABZ is a weakly basic drug and its solubility is pH-dependent, the release rate from the EuRS polymer-coated TAP cores at pH 1.2 was high, reaching approximately 60% of the total drug released within the first 2 h (Figure 5c). TAP-EuRS10% pellets exhibited a more pronounced release compared to the other two non-pH-modifier cores with the same layering structure at pH 1.2 (Figure 5a,b). The higher release can be the result of the higher osmotic and/or microenvironmental effect. It was previously described that the addition of pH modifiers increased the drug release also at an acidic pH (pH = 1.2), and the extent of the increase depended on the type and concentration of the acid applied [43]. However, unlike tartaric acid, when fumaric acid was used as a pH modifier in a study by Gutsche et al., the verapamil hydrochloride release from alginate matrix tablets was not very high in acidic-pH dissolution medium, which was explained by the low solubility of fumaric acid in pH 1 [44]. Although both acids have similar pKa values (2.93 and 4.23 for tartaric acid; 3.03 and 4.54 for fumaric acid [41]), their solubility in water differs significantly. Tartaric acid has much higher solubility: in water, at a temperature of 25 °C, the solubility is 1395.8 g/L for tartaric acid and 6.6–8.1 g/L for fumaric acid [45]. In a study by Guthmann et al., the lower solubility of fumaric acid slowed down its release from a formulation, compared to tartaric acid, maintaining a constant microenvironmental pH within the pellet, which provided higher drug release at a pH 6.8 buffer solution [46]. Our results show that tartaric acid pellet cores can also improve the release of a weakly basic drug at a higher pH. Still, the application of an enteric coating is needed to protect its release from the formulation.

The results with sugar and MCC align with the observation of Kállai et al., who noted that sugar cores resulted in a higher release rate, compared to MCC, due to their osmotic effect [31].

To prevent the release of ABZ at pH 1.2 and enhance its release at higher pH values, a pH-dependent polymer was applied as an external coating layer. Compared to EuRS, for which the release was already high at the beginning at acidic pH, the application of EuFS almost completely prevented release at pH 1.2, decreasing the cumulative drug release at pH 6.8.

Contrary to EuFS, at pH 6.8, EuRS regulates the dissolution medium penetration to the system, which leads to a decrease in the pH in the core of the coated pellet (in the first 30 min; Figure 6). The decrease is caused by the dissolution of tartaric acid in the medium that entered the formulation (Figure 7c). However, as this layer also regulates the release of tartaric acid from the formulation, an increase in the microenvironmental pH occurs. The rapid increase in the microenvironmental pH was confirmed by the rapid color change seen in the pellet and the medium induced by the indicator in the microfluidic cell, indicating the rapid dissolution and release of tartaric acid from the pellets. The high release of tartaric acid has been previously described for 10% Eudragit^®^ RS films at a basic pH [47].

The effect of microenvironmental pH is possible if a pH-modifier does not leach out from the formulation prematurely and remains inside in an adequate amount to provide the acidic pH during drug release [48]. In the case of TAP-EuRS10%-EuFS25%-layered pellets, the color change seen in the microfluidic cell started after a lag time and was more pronounced in the center of the pellet than in the medium. These findings suggest that tartaric acid is not readily released from the EuRS-EuFS coating at pH levels lower than 7 (pH = 6.8). Therefore, the application of the EuFS layer not only prevented the release of albendazole at a lower pH, characteristic of the higher parts of the gastrointestinal tract, but also protected tartaric acid from its release at pH 6.8 to a greater extent than EuRS, enhancing the pH-modifier effect of TAP. This finding is in agreement with the study by Ploen et al., where an enteric coating was applied to control the release of citric acid from the pellet core and prevent its rapid release from the formulation [48]. The decrease in pH inside the coated pellet increases ABZ solubility and release in a higher pH dissolution medium. These findings further confirm the hypothesis that the enteric-coated TAP pellets have a more efficient pH-modifier effect compared to the cores that are layered with a time-dependent layer polymer only. The decreased microenvironmental pH in the case of the presence of the pH modifier in the formulation was also observed in previous studies. When fumaric acid was added to a drug–alginate matrix system, the lower pH of frozen and subsequently cut cryosections was determined using a surface pH electrode, compared to alginate–matrix tablets without the organic acid, where the pH was higher [44]. Streubel et al. showed that the addition of organic acid to the matrix tablets provided a pH-independent release of a weakly basic drug, because of the acidic microenvironmental pH, which was maintained due to the amount of the organic acid that remains in the formulation [49].

EuFS is an anionic polymer with carboxylic groups that ionize in a neutral-to-alkaline medium. The pH of the ileum ranges from 6.6, reaching about 7.5, then decreases in the ascending colon to pH 6.4, and rising again in the right colon to a pH of up to 7 [50,51]. Therefore, the release from pH-dependent polymers that dissolve above 7 can be classified as ileocolonic delivery [52]. However, the colon delivery formulations should release more drug in the colon and less in the small intestine [53]. Our results highlight that, to maximize ABZ release at the higher pH of the gastrointestinal tract, the combination of pH-dependent and time-dependent polymers is preferable (Figure 5d). This aligns with Handali et al., who suggest that time-dependent polymers offer more reliable release by delaying it until the formulation reaches the colon [53]. Using EuRS together with EuFS, compared to EuRL-EuFS and EuFS alone, resulted in a more significant delayed release and minimized release at lower pH levels (Figure 5d).

In a previous study, Siepe et al. investigated the relationship between the type and characteristics of organic acid and its release from the formulation, thereby affecting the duration of the microenvironmental pH [54]. Guthman et al. revealed that when pellet cores containing tartaric acid were used, the release remained pH-dependent, suggesting the limited use of tartaric acid pellet cores as a pH modifier because of its high solubility and lower acidic strength [46]. However, our findings provide evidence that, when applied as functional starter cores in enteric-coated multiparticulate dosage forms, tartaric acid pellets increase the release of ABZ at higher pH levels compared to the use of non-pH-modifier cores. These results are in agreement with the study by Pareek et al., who demonstrated that InstaSpheres TA containing tartaric acid can be used as pH-modifier cores for a weakly basic drug [25].

**Figure 10:**
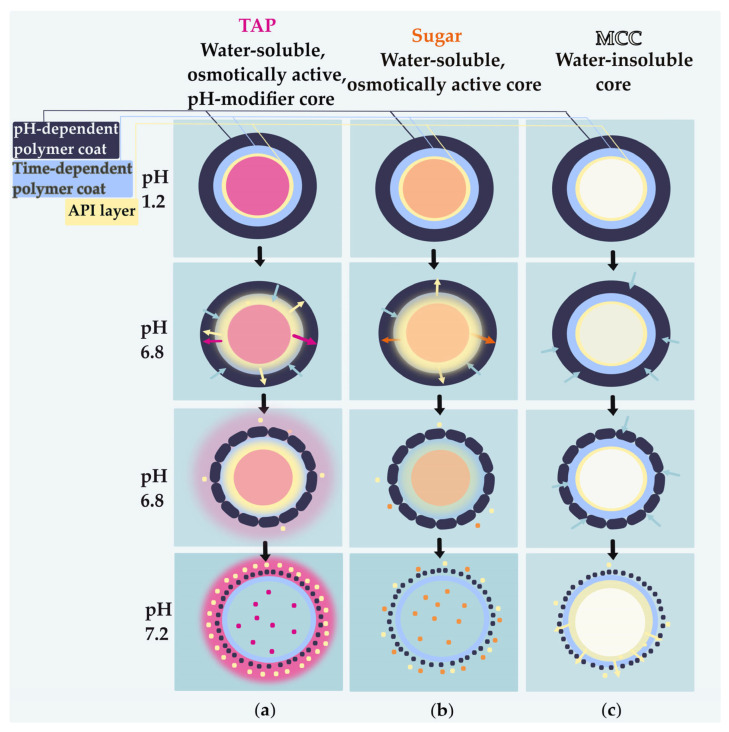
Schematic illustration of ABZ release from time- and pH-dependent polymer-coated (**a**) TAP, (**b**) sugar, and (**c**) MCC pellets (2 h at pH 1.2, then 4 h at pH 6.8, and further 18 h at pH 7.2). The dots represent the ABZ or excipients, the arrows show the direction of the material transfer.

The schematic drug-release mechanism from the three types of coated pellets is illustrated in Figure 10. At pH 1.2, the drug release was practically completely prevented for all three types of cores. After 2 h, the pH was changed to 6.8, at which point the drug release started to increase slightly by TAP and minimally by sugar pellets. The release of the drug from EuFS at a pH lower than 7 can be attributed to the microenvironmental and/or osmotic effects of TAP. At a pH of 7.2, the drug is completely released. The pH-modifier effect of the core enhances the release from TAP-coated pellets. In the case of sugar pellets, the release of the drug is driven by the osmotic force of sugar dissolving upon the entrance of the dissolution medium inside the formulation, and the release remains lower compared to TAP.

In contrast, the release from MCC is very low over 24 h, which can be explained by the fact that MCC is a water-insoluble core type. Compared to sugar, MCC cannot dissolve in the dissolution medium that enters the pellet. Therefore, no osmotic pressure can be generated to promote drug release. The inert pellet cores can influence the drug-release process. The TAP cores of high solubility and disintegration influence the release of active ingredients from the coated-pellet delivery systems due to the dissolved tartaric acid performing both a microenvironmental pH change towards an acidic region, where the solubility of ABZ is increased, and an osmotic effect. In the osmolality studies conducted, the TAP solution exhibited the highest osmolality, compared to the solution of sugar spheres or water-insoluble MCC.

## 5. Conclusions

Tartaric acid beads, utilized as core functional starters, are capable of sustaining a localized acidic microenvironment within the coated pellets, facilitating increased solubility and release of albendazole from enteric-coated pellets under elevated pH conditions. Unlike formulations employing solely time-dependent polymers—where tartaric acid release is accelerated at a higher pH depending on the polymer coating’s water permeability—the additional application of an EuFS layer effectively inhibits premature leaching of tartaric acid, thereby preserving the acidic microenvironmental pH. The combination of the time-dependent EuRS polymer with the pH-responsive EuFS polymer was preferred over EuRL-EuFS or EuFS alone, as it produced a more substantial delayed-release profile, thus more reliably preventing drug release in the upper gastrointestinal tract. Enhanced albendazole release from tartaric acid pellet (TAP)-based, drug-loaded polymer-coated pellets was observed compared to pellets with identical layering but inert sugar or microcrystalline cellulose (MCC) cores. Overall, this study demonstrates the potential of ready-to-use, 100% tartaric acid pellets to improve the solubility of the weakly basic drug albendazole, particularly enhancing its dissolution at the ileocolonic target site. Tartaric acid beads functioning as microenvironmental pH modifiers have the potential to be utilized in conjunction with polymer film coatings applied to other BCS class II–IV drugs.

## Figures and Tables

**Table 1:** Layering and coating conditions.

	I.	II.		III.
		a	b	
Process	ABZ Layering	EuRS Coating	EuRL Coating	EuFS Coating
Batch size (g)	600	200	200	150
Spray-nozzle diameter (mm)	0.8	0.8	0.8	1.2
Set temperature (°C)	50	50	50	25
Inlet air temperature (°C)	49	44–45	44–45	22
Outlet air temperature (°C)	41	35–36	35–36	18
Air pressure (bar)	0.8	0.8	1.0	1.0
The capacity of the fan	2–3	2–3	4	2
Feeding rate (g/min)	2.0–3.0	3.0	3.0	3.7
Drying temperature (°C)	45	45	45	22
Dry polymer increase (%)	0.6	10	10	15.6
Weight increase (%)	4.1	19.5	19.5	25.8

**Table 2:** Spearman’s rank correlation coefficient between ΔE* (medium) and ΔE* (pellet), and the % drug release from TAP-EuRS10% and TAP-EuRS10%-EuFS25% at pH 6.8 at specific time points.

		Cumulative Drug Release (%)	
		TAP-EuRS10%	TAP-EuRS10%-EuFS25%
ΔE*	Medium	0.91	0.81
	Pellet core	0.91	0.86

## Data Availability

Data is contained within the article or Supplementary Material.

## Supplementary Materials

The following supporting information can be downloaded at: https://www.mdpi.com/article/10.3390/pharmaceutics17091133/s1, Table S1: Size and morphology parameters of cores and layered, coated pellets; Table S2: The colour change (ΔE*) observed in the pellet core and the surrounding medium in the case of TAP-EuRS10% and TAP-EuRS10%-EuFS25% layered pellets.

## Abbreviations

The following abbreviations are used in this manuscript:

ABZ	Albendazole
API	Active pharmaceutical ingredient
BCS	Biopharmaceutical classification system
EuRS	Eudragit^®^ RS
EuRL	Eudragit^®^ RL
EuRS-EuFS	Eudragit^®^ RS-Eudragit^®^ FS
EuRL-EuFS	Eudragit^®^ RL-Eudragit^®^ FS
EuFS	Eudragit^®^ FS
H	Hour/hours
HPMC	Hydroxypropyl methylcellulose
IC50	Half-maximal inhibitory concentration
MCC	Microcrystalline cellulose
TAP	Tartaric acid pellets
TNF	Tumor necrosis factor
