# Supplementary material for: Microenvironmental pH-Modulated Dissolution of Albendazole Layered on Tartaric Acid Starter Pellet Cores
Source: Pharmaceutics. 2025 Aug 29;17(9):1133. doi: 10.3390/pharmaceutics17091133 (PMC12474003; doi:10.3390/pharmaceutics17091133)
Supplement: Supplementary file 1 [file pharmaceutics-17-01133-s001.zip › pharmaceutics-3821419-supplementary.pdf]

Supplementary Materials: Microenvironmental pH-Modulated Dissolution of Albendazole Layered on Tartaric Acid Starter Pellet Cores

Kristina Vlahovic[1†], Miléna Lengyel[1†], Christian Fleck[1], Nikolett Kállai-Szabó[1,2], Emese Balogh[1], András József Laki[3,4] and István Antal[1,2*]

Table S1. Size and morphology parameters of cores and layered, coated pellets

Core type	Core/ core+coating layer	Area (mm²)	$Feret_{max}$ (mm)	$Feret_{min}$ (mm)	Diameter (mm)	AR	Roundness
TAP	core	0.40±0.06	0.82±0.07	0.70±0.05	0.71±0.05	1.15±0.09	0.87±0.07
	HPMC (ABZ)	0.42±0.07	0.84±0.09	0.70±0.06	0.73±0.08	1.15±0.08	0.87±0.06
	EuRS10%	0.48±0.06	0.88±0.07	0.75±0.05	0.78±0.05	1.15±0.09	0.87±0.06
	EuRL10%	0.50±0.04	0.88±0.05	0.76±0.03	0.80±0.05	1.13±0.08	0.89±0.05
	EuFS25%	0.53±0.03	0.91±0.01	0.73±0.03	0.82±0.03	1.15±0.09	0.87±0.05
	EuRL10-EuFS25%	0.58±0.06	1.04±0.03	0.82±0.04	0.86±0.04	1.14±0.09	0.88±0.06
	EuRS10%-EuFS25%	0.56±0.08	0.96±0.08	0.81±0.07	0.85±0.06	1.15±0.10	0.87±0.06
MCC	core	0.34±0.05	0.70±0.07	0.63±0.04	0.65±0.05	1.09±0.08	0.92±0.05
	HPMC (ABZ)	0.36±0.05	0.71±0.05	0.65±0.02	0.67±0.05	1.09±0.07	0.92±0.05
	EuRS 10%	0.40±0.07	0.76±0.09	0.69±0.05	0.71±0.05	1.09±0.07	0.92±0.05
	EuRS10% EuFS25%	0.45±0.06	0.82±0.07	0.73±0.05	0.75±0.05	1.08±0.06	0.99±0.05
Sugar	core	0.56±0.06	0.91±0.06	0.81±0.05	0.84±0.05	1.10±0.07	0.91±0.06
	HPMC (ABZ)	0.59±0.08	0.94±0.10	0.83±0.04	0.86±0.05	1.11±0.15	0.91±0.07
	EuRS 10%	0.65±0.06	1.01±0.06	0.89±0.04	0.91±0.04	1.09±0.05	0.92±0.04
	EuRS10% EuFS25%	0.75±0.06	1.05±0.06	0.95±0.05	0.98±0.05	1.08±0.05	0.92±0.04

Table S2. The colour change (ΔE*) observed in the pellet core and the surrounding medium in the case of TAP-EuRS10% and TAP-EuRS10%-EuFS25% layered pellets

**time (min)**	**ΔE*** $_{\mathbf{EuRS10\%}}$		**ΔE*** $_{\mathbf{EuRS10\%\text{-}FS25\%}}$	
	medium	pellet	medium	pellet
**5**	0.24±0.18	3.43±0.48	0.63±0.15	0.89±0.10
**15**	4.48±1.03	14.34±0.60	0.76±0.12	1.88±0.21
**30**	23.71±1.38	39.90±4.09	0.77±0.22	5.45±0.87
**45**	26.27±1.63	42.28±4.55	1.07±0.26	6.50±0.78
**90**	24.26±1.60	42.58±3.34	2.28±0.39	19.50±1.12
**120**	23.22±1.46	39.28±1.21	19.91±1.49	39.44±2.16
**150**	24.52±1.16	40.09±0.65	26.35±1.71	51.47±4.43
